# Oysters and Typhoid: Proposed Legislation

**Published:** 1896-05-23

**Authors:** 


### OYSTERS AND TYPHOID: PROPOSED LEGISLATION.

It pleases a portion of the public to think that the medical profession is given to the making of "scares."

But there was something in the "oysters and typhoid" alarm after all. Those medical men who considered the source from which it emanated —Sir William Broadbent was its sponsor—always knew there was. At the present moment so important a town as Brighton is agitating for protective legislation, as the result of its own experience. According to evidence given before the Select Committee of the House of Commons last week, the oyster beds at Southwick and Shoreham Harbour have "produced a large number of cases of typhoid fever within the last three years." If such be the case, then it is clear that Brighton, in its own interests, must find means of protecting its inhabitants, and its visitors, from such very serious dangers. Those strangers who go to Brighton go there mostly for health; and among convalescents and others in search of health good oysters are if not a daily necessity certainly a daily pleasure.

There cannot, then, be two opinions as to the necessity on the part of Brighton for taking such measures, whatever they may be, as will effectually protect both her inhabitants and her visitors against any danger whatever from the eating of oysters. But if the oyster beds at Brighton are liable to be dangerously contaminated by sewage the presumption is that other oyster beds in other localities may be subject to the same liability. In like manner, if legislation be necessary for Brighton, it is fair to suppose that it is necessary for other localities. It is pretty clear, then, that what is wanted is not a local Act but a general Act, elastic enough to be locally applied.

The Local Government Board appeared by counsel as an opponent of the Brighton scheme; and we think wisely. The Corporation of Brighton asks for powers of a somewhat drastic kind—powers which would enable it, in case of necessity, compulsorily to prohibit the use of any particular oyster beds, and so to destroy the property of individuals. No doubt such powers should be given to some public body, but the question is, to what body? The Local Government Board declares that such powers ought to be central and not local, and ought to be administered by persons who cannot be influenced one way or another by merely local considerations. Therein the action of the Local Government Board must command general approval.

What is wanted, not only at Brighton, but everywhere else where oysters are produced, is inspection, and inspection which will be thoroughly efficient. At Brighton itself, and similar places, where probably most of the oysters produced are consumed on the spot, and where a considerable production of bad oysters would mean a very heavy money loss to the whole town, it is highly probable that great efforts would be made to render inspection entirely efficient. But at other places, where there is no great local consumption, and where, instead, most of the oysters produced are packed and sent to a distance, where is the adequate local motive for effective inspection? There is no such incentive. On the contrary, both proprietors and workers would be directly interested in keeping the cost of production at the lowest possible level, and the most powerful motive prevalent all round would be the motive of resisting interference. It seems clear, then, that if oyster legislation is to be serviceable to London as well as to Brighton, to the whole country as well as to one particular town, it must provide for adequate inspection by means of independent inspectors who have no local "axes to grind" of any sort.

There are, no doubt, some persons who think that the whole question savours of "grandmotherly legislation," and that such a business as the oyster industry might very well have been left to take care of itself. The more obvious aspect of the case is, however, this: that at a period when scientific food inspection has been carried to great lengths, when not only meat markets and fish markets, but vegetable markets and private dealers' shops of all kinds, are the subjects of inspection, the fact that so very considerable a source of perishable food supply as our coast oyster beds should have been left with practically no inspection for so many years is a remarkable circumstance, and as unsatisfactory as it is remarkable. What legislation will do and ought to do is simply to bring in from the outlying and, so far, uncivilised regions of no scientific inspection a very large, very important, and often very dangerous source of food supply. This is clearly as it should be; and every person who is in sympathy with knowledge as against ignorance and with science as against dangerous and often deadly darkness, will hope that adequate legislation for securing the plentiful production of fat and wholesome oysters will be carried through without a moment's unnecessary delay.

